# Exploring patient activation and self-management experiences in adults with fibromyalgia: a qualitative evidence synthesis

**DOI:** 10.1093/rap/rkaf025

**Published:** 2025-03-10

**Authors:** Kit Yung, Durva Jadhav, Cheuk Ma, Sakshee Majgaonkar, Eya Manai, Jennifer Pearson

**Affiliations:** School of Health and Social Wellbeing, University of the West of England, Bristol, UK; School of Health and Social Wellbeing, University of the West of England, Bristol, UK; School of Health and Social Wellbeing, University of the West of England, Bristol, UK; School of Health and Social Wellbeing, University of the West of England, Bristol, UK; School of Health and Social Wellbeing, University of the West of England, Bristol, UK; School of Health and Social Wellbeing, University of the West of England, Bristol, UK; The RNHRD and Brownsword Therapies Centre, Royal United Hospital Bath, Bath, UK

**Keywords:** fibromyalgia, self-management, patient activation, qualitative

## Abstract

**Objectives:**

Fibromyalgia syndrome (FMS) is a chronic pain condition that affects involvement in daily activities, including self-care and household responsibilities. Self-management strategies are a primary focus in treatment recommendations. However, their effectiveness depends on an individual’s readiness and capacity to adopt health-promoting behaviours. This study aims to explore the experiences of adults in their self-management journey, focusing on the barriers and facilitators influencing patient activation (PA) and effective self-management.

**Methods:**

A qualitative evidence synthesis was conducted. An electronic search was performed using the following databases: CINAHL, PsycINFO, PubMed, Medline, ScienceDirect and AMED. The studies were screened against eligibility criteria to ensure their relevance. The quality of the included studies was assessed against the Critical Appraisal Skills Programme (CASP) questionnaire for qualitative studies and the Consolidated Criteria for Reporting Qualitative Research (COREQ) checklist. Findings from the papers were synthesized via the three-stage thematic synthesis process, and common themes were identified.

**Results:**

Nine studies with a total of 130 participants were included. Four major analytical themes were identified, including legitimizing FMS, the value of medical support, receiving peer and social support, and learning to self-manage.

**Conclusion:**

Self-management of FMS requires patients to be actively involved in managing their health. These findings highlight that support from HCPs, family members and peers helps patients learn how to self-manage and engage in health-promoting behaviours. Clinicians treating people with FMS should prioritize education, empathy and personalized support.

Key messages

- Adequate support from the biomedical field was a prominent factor in facilitating the development of patient activation and thus benefit self-management.
- Consequences of physical activities, such as pain and fatigue, are often viewed by patients as barriers to further engagement with exercise programmes and reduce their willingness to exercise.
- Due to the invisibility and invalidation of fibromyalgia syndrome, there is a need for legitimization and diagnostic clarification for this population of patients.

## Introduction

Fibromyalgia syndrome (FMS) is a complex chronic condition characterized by widespread musculoskeletal pain and fatigue, which commonly affects one’s ability and involvement in functional activities [1]. It is estimated that approximately 5.4% of the population in the UK is affected by FMS, with a higher prevalence in women than men [2]. FMS is often described an ‘invisible illness’, as it lacks visible physical symptoms, causing those affected to explain and legitimize their condition to others [3, 4].

According to current guidelines, implementing self-management strategies has been shown to optimize patients’ functioning and quality of life [5, 6]. However, research has indicated that patient outcomes and adherence to self-management are commonly dependent on concepts such as one’s readiness to change, self-efficacy or PA [7–10]. The self-efficacy theory developed by Bandura [11] defined self-efficacy as an individual’s belief in their ability to perform actions, which may influence adaptive coping behaviours in rehabilitation. PA, however, draws upon self-efficacy and readiness to change, capturing elements of each and forming a more generalized concept that refers to a person’s knowledge, skills and confidence in managing their health [12, 13]. This allows for a more nuanced understanding of individual’s capacity to manage their own health.

PA facilitates treatment adherence and health-promoting behaviours in individuals with chronic conditions [14]. Positive correlations between PA and functional capacity were found in the field of inflammatory arthritis (IA) [15, 16]. A recent study by Jones *et al*. [17] explored the factors influencing PA in individuals with IA and found that self-efficacy and health literacy were significantly associated with PA. Additionally, a study by Yao *et al*. [18] identified the sociodemographic characteristics of participants—those with lower activation levels often had lower educational levels and were employed in manual occupations. However, due to their broad focus on chronic pain, the sample was heterogeneous, with patients presenting with cancer pain and other co-morbidities.

While studies have focused on understanding the illness experience and self-management in people with FMS, limited research has systematically explored PA in FMS specifically [19, 20]. The invisibility, diagnostic challenges and invalidation of FMS distinguishes it from other conditions [21–24]. Therefore, by systematically reviewing current qualitative literature, this study explores the barriers and facilitators influencing PA and, thus, self-management in adults with FMS.

## Methods

### The qualitative approach

A qualitative evidence synthesis (QES) was conducted. This review is reported in compliance with the preferred reporting items for systematic reviews and meta-analyses (PRISMA) statement [25]. This work formed the basis of an undergraduate dissertation, with protocol submitted for internal academic review. The processes were conducted with triangulation to optimize the findings’ credibility [26].

### Search strategy

The following databases were searched—AMED, PubMed, CINAHL, Medline, ScienceDirect and PsycINFO. The search terms were developed based on the SPIDER framework, alongside synonyms, to allow a greater breadth of search results [27]. However, after initial trials of searching, including ‘patient activation’ in the search terms and ‘research type’ related words such as ‘qualitative’ limited search results. Therefore, meetings were held with a librarian and optimized the search terms to yield the most results (Table 1). Moreover, snowballing was performed to review reference lists of included studies and search for missing seminal papers [28].

**Table 1.** Search terms

Database	SPIDER tool	Search terms
AMED, PubMed, CINAHL, Medline and PsycINFO	Sample	‘fibromyalgia’ OR ‘chronic pain’ OR ‘fibro*’ OR ‘fibromyal*’ OR ‘chronic MSK pain’
	Phenomenon of interest	‘self-management’ OR ‘self care’ OR ‘self-manage*’
	Design	‘interview’ OR ‘focus groups’ OR ‘semi-structured interview’
	Evaluation	‘behavi* change’ OR ‘adhere*’ OR ‘patient compliance’ OR ‘patient experience’
ScienceDirect	As above	(‘fibromyalgia’ OR ‘chronic msk pain’) AND (‘self-management’ OR ‘self-care’) AND (‘interview’ OR ‘focus groups’) AND (‘behaviour change’ OR ‘patient experience’ OR ‘adhere’)

### Screening process, inclusion and exclusion criteria

Two pairs of reviewers (K.Y. and S.M.) and (D.J. and C.M.) independently screened title and abstract followed by a manual assessment based upon of inclusion and exclusion criteria (Table 2). Eligible studies references were exported to Mendeley Desktop (version 1.19.8) for duplicate removal. Decisions were made through group discussions, with a third reviewer (J.P.) consulted when a consensus was not reached.

**Table 2.** Inclusion and exclusion criteria

Inclusion	Exclusion
Only English papers	Papers not in English
Qualitative research studies	Quantitative research studies, systematic literature reviews and grey literature
Study population includes only adults with fibromyalgia	Papers including heterogeneous chronic pain or musculoskeletal conditions
Date of studies (2000–present, i.e. 2023)	Papers published before 2000
Interventions used in studies are self-management techniques (i.e. physical activity and symptom management)	Interventions used in the studies are not self-management techniques
Outpatient and community setting	Inpatient setting

#### Type of studies

With our research focus, only primary qualitative studies were included to produce integrated findings to enrich the understanding towards PA and self-management in FMS. Grey literature was excluded, considering the difficulty in retrieving this type of study [29]. Due to the evolution of the understanding towards FMS in the biomedical field, older studies may have focused on outdated interventions and concepts [30]. Therefore, studies published before the year 2000 were also excluded. Due to the unique nature of FMS and the label it carries, studies including other conditions were excluded to enable a closer inspection on the impacts of PA in self-managing FMS. Moreover, only studies in English were included due to the lack of translators.

#### Self-management techniques and interventions

We excluded studies that did not include any elements of self-management or conducted in in-patient settings where patients are managed by healthcare professionals (HCPs). Studies focusing either on self-management interventions led by HCPs or strategies implemented by patients were all included.

#### Relevance to PA

Moreover, since the key focus PA was not included in the search terms, the constructs of PA (knowledge, skills and confidence) were screened during the full-text eligibility assessments, and studies irrelevant to those constructs were excluded.

### Quality assessment

The Critical Appraisal Skills Programme (CASP) tool for qualitative studies was applied for quality assessment [31]. As recommended by Cochrane, CASP assesses the methodological rigour and limitations of studies [32–35]. To better conceptualize quality and enable comparison, each question was scored 0 to 2 [36], where 0 = not answered, 1 = somewhat answered and 2 = well answered. Scoring was conducted independently by two pairs of researchers (K.Y. and C.M.) and (D.J., S.M. and E.M.). Limitations were identified by highlighting the scores below 2. In addition to methodological rigour, reporting can affect quality [37]. Therefore, reporting transparency was assessed using the Consolidated Criteria for Reporting Qualitative Research (COREQ) framework [38].

### Data extraction

Characteristics of the included studies were manually extracted and formed a table (Table 3). The process was conducted by two pairs of reviewers (C.M. and K.Y.) and (D.J., S.M. and E.M.) independently. Characteristics of participants were also extracted to inform demographics among included studies. Qualitative data were extracted from the results sections and imported into NVivo (version 20.7.1) for synthesis.

**Table 3.** Data extraction table

#	Author (country)	Data collection method	Participants	Self-management components
1	Arfuch *et al*., 2022 (USA)	Semi-structured interview	*N* = 10 (M: 0, F: 10), mean age: 58.5, mean duration of FMS: 11 years (min 2, max 30)	Pain management, physical activity, emotional management, nutrition, insomnia management
2	Chen, 2016 (USA)	Interview	*N* = 23 (M: 1, F: 22), age range: 21–79 years, illness duration: 1–58 years	Pain management, emotional regulation, physical activity, nutrition, self-education
3	Lempp *et al*., 2009 (UK)	Semi-structured interview	*N* = 12 (M: 1, F: 11), mean age: 49, mean duration of FMS: 3 years (min 5 months, max 11 years)	Overall experience on FMS impact on daily life, general overview on self-management
4	McIlroy *et al*., 2022 (UK)	Semi‐structured telephone interview and focus groups	*N* = 13 (6: HCP, 7: patient participants) Patient participants: M: 0, F: 7, mean age: 46	Pain management, emotional management, physical activity
5	Rasmussen *et al*., 2017 (Denmark)	Semi-structured focus group interviews	*N* = 17 (M: 0, F: 17), mean age: 42.8, median duration of FMS: 11 years (min 6 months, max 12 years)	Physical activity, pain management, sleep hygiene
6	Russell *et al*., 2018 (Ireland)	Focus group	*N* = 14 (M: 2, F: 12)	Fatigue and insomnia management, physical activity, therapeutic exercises
7	Sallinen *et al*., 2011 (Finland)	Narrative interview	*N* = 20 (M: 0, F: 20), mean age: 54 years, pain duration: 10–30 years	Overall coping with fibromyalgia, general view on self-management
8	Kengen Traska *et al*., 2011 (USA)	Qualitative descriptive study with group interviews	*N* = 8 (M: 0, F: 8), mean age: 60 years, illness duration: > 6 years	Overall view on self-management including symptom management, pacing techniques, emotional regulation
9	Pearson *et al*., 2020 (UK)	Non-participatory observations followed by semi-structured interview	Observations: *N* = 21 (M: 2, F: 19), interview: *N* = 13 (4 therapists, 9 patients, all female)	Physical activity, therapeutic exercises, pacing techniques, goal setting, emotional regulation, nutrition, sleep hygiene

FMS: fibromyalgia syndrome; HCP: healthcare professional.

### Data synthesis

Recommended by Cochrane Training, thematic synthesis was applied through a three-step process [39]. Starting with line-by-line coding, thematic synthesis aids the development of ‘descriptive themes’ based on commonalities within codes, and generating ‘analytical themes’ that address the research topic [40]. Inductive line-by-line coding was conducted using NVivo to form a new framework from the findings. The results sections of each study were coded by two pairs of reviewers independently (C.M. and D.J.) and (K.Y., S.M. and E.M.). An iterative approach was applied when forming themes and re-visiting the raw qualitative data to identify commonalities. The processes of theme development were performed among the whole team, and all agreed on the final analytical themes.

### Reflexivity

Team governance procedures were conducted to minimize individual influence on the interpretation of results [41]. All key stages were conducted by two pairs of reviewers independently, and differences in findings were discussed as a group. To ensure a comprehensive interpretation of the results, researchers with different backgrounds were paired [42]. As physiotherapy students, K.Y., D.J., C.M., S.M. and E.M. consulted J.P., an experienced clinical academic with expertise in FMS, before making key decisions. An audit trail of major decisions was kept throughout all stages of the research process [41].

## Results

### Study selection

**Figure 1.**
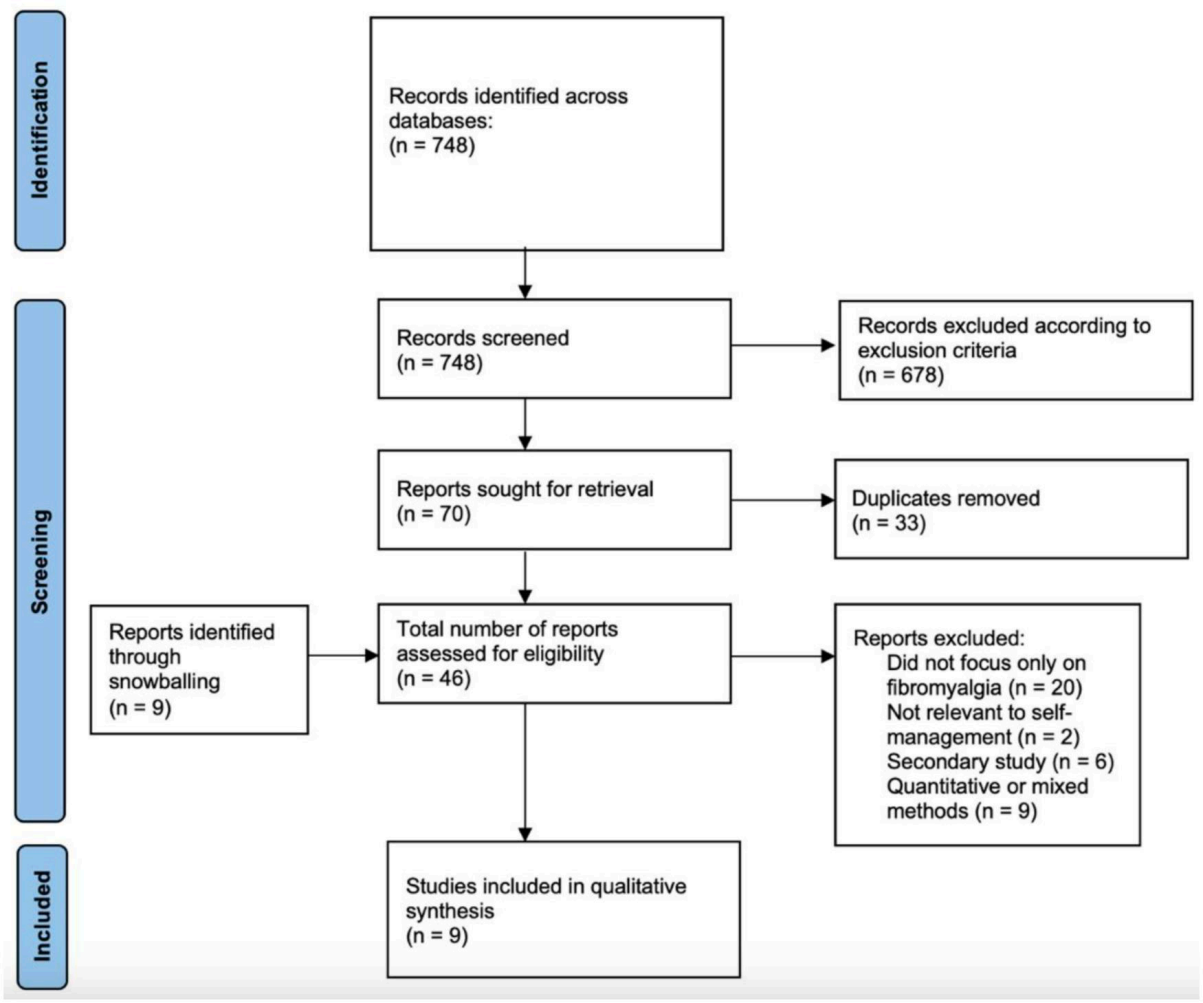
Preferred reporting items for systematic reviews and meta-analyses (PRISMA) flow diagram

A total of 748 studies were yielded across all databases. After title and abstract screening, 70 papers were sought for retrieval. After removing duplicates and including nine papers identified from snowballing, the eligibility of these papers was assessed via full-text screening, which resulted in nine papers meeting the full inclusion criteria (Fig. 1).

### Study characteristics

Of the nine papers, two included both HCP and patient participants [41, 43], while the remaining seven recruited patients only, with a total of 120 patients and 10 HCPs. Patients were mostly female (four males), aged 20–73, and their illness duration ranged from 6 months to 58 years. Four studies targeted their study towards the experiences of specific intervention programmes [43–46], while the other five studies aimed to understand the general experience in self-managing FMS [47–51] (see Table 3).

### Quality assessment

The results of the CASP appraisal tool and COREQ checklist were included (see Supplementary Tables S1 and S2, available at *Rheumatology Advances in Practice* online). In the CASP appraisal, all papers scored 17/20 or above. However, poor reporting in several papers was revealed in COREQ despite achieving a high score on CASP [47, 48, 50, 51]. Items regarding the reasons for non-participation, the presence of repeat interviews and the description of coding trees were scarcely reported. Only two papers specified the gender of the researchers, knowing that in a predominantly female sample, gender-related bias may be present [44, 50].

### Synthesis of results

**Figure 2.**
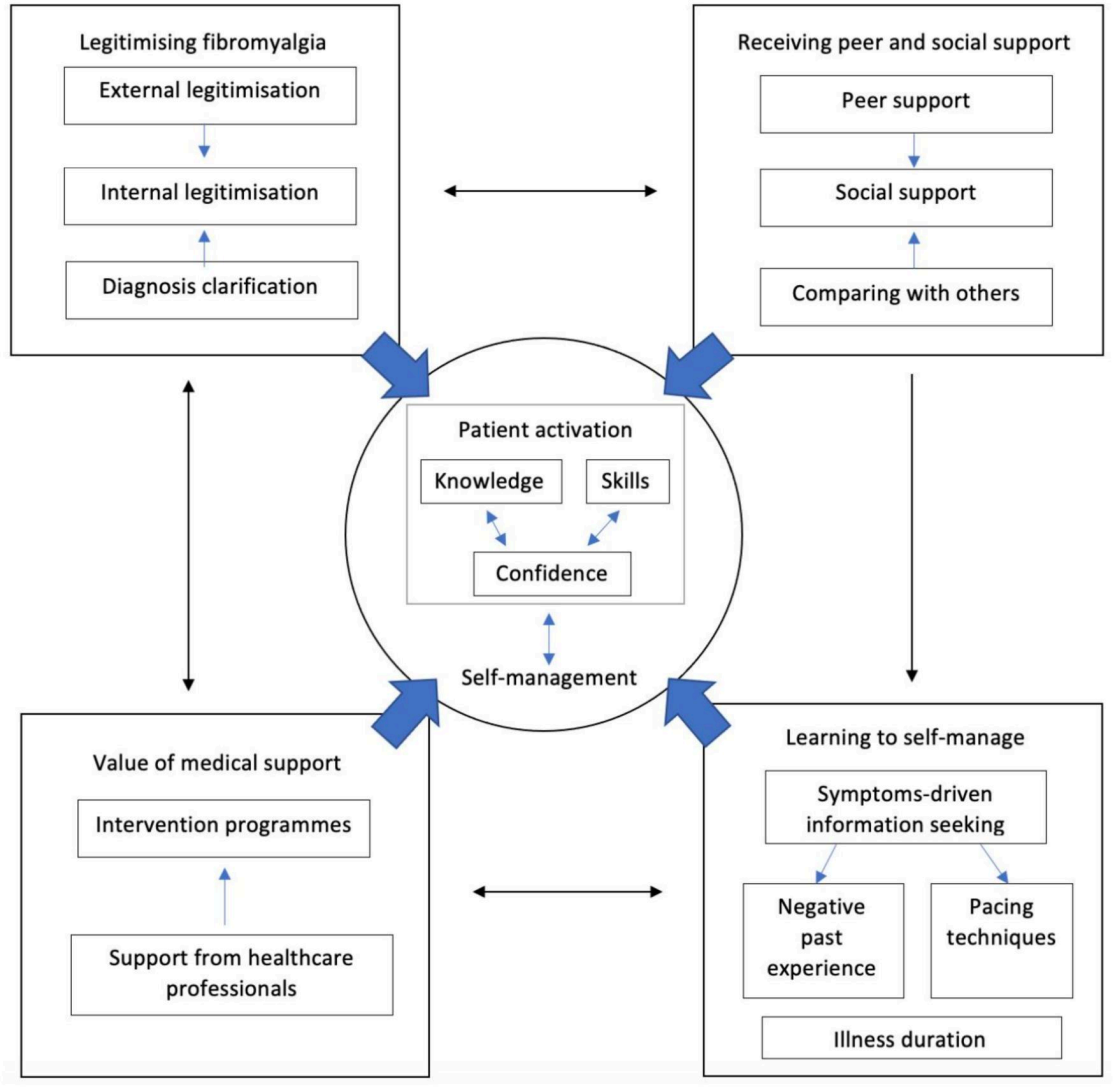
Framework illustrating the interrelationships between themes and how they contribute to PA and self-management

Four main analytical themes emerged from descriptive themes—‘legitimizing FMS’, ‘value of medical support’, ‘receiving peer and social support’ and ‘learning to self-manage’ (see Fig. 2). While the themes ‘legitimizing FMS’ and ‘learning to self-managed’ derived solely from patients’ perspectives, the other two themes comprised both HCPs and patients’ narratives. Supporting quotes for each subtheme are in Supplementary Table S3, available at *Rheumatology Advances in Practice* online, with participant quotes marked as ‘P’ and author interpretations marked as ‘A’. See Table 4 for the contribution of studies to the synthesis.

**Table 4.** Contribution of studies to the qualitative synthesis

Analytical theme	Descriptive theme	Arfuch *et al*. (2022)	Chen (2016)	Lempp *et al*. (2009)	McIlroy *et al*. (2022)	Rasmussen *et al.* (2017)	Russell *et al*. (2018)	Sallinen *et al*. (2011)	Kengen Traska *et al*. (2011)	Pearson *et al*. (2020)
Legitimizing FMS	Internal legitimization	✓ ✓	✓ ✓			✓ ✓	✓		✓	
	External legitimization	✓ ✓		✓		✓	✓	✓ ✓		
	Value of diagnosis		✓ ✓	✓ ✓		✓ ✓				
Receiving peer and social support	Peer support	✓ ✓				✓ ✓	✓	✓ ✓	✓	
	Social support			✓ ✓		✓ ✓	✓		✓	
	Comparing with others	✓ ✓						✓ ✓		
Value of medical support	Intervention programmes	✓ ✓			✓ ✓	✓ ✓				✓ ✓
	HCP support	✓ ✓	✓	✓	✓ ✓	✓ ✓	✓ ✓	✓	✓	✓
Process of learning to self-manage	Illness duration		✓ ✓		✓				✓ ✓	
	Symptoms-driven information seeking		✓ ✓	✓			✓	✓		
	Negative past experiences			✓			✓ ✓			
	Pacing techniques					✓ ✓	✓		✓	✓ ✓

Double ticks ‘✓ ✓’ represent evidence supporting the link between the subtheme and both PA and self-management, while one tick ‘✓’ represents the link between the subtheme and self-management only.

FMS: fibromyalgia syndrome; HCP: healthcare professional.

#### Legitimizing FMS

Due to the invisibility and unpredictability of FMS, many people living with FMS were viewed as ‘invalid’ by others and experienced a low acceptance and legitimacy in their illness accounts [43, 47–50]. Therefore, external legitimization and acceptance from others were highly valued and were shown to improve internal legitimization [43, 48, 49, 51]. External legitimization is often achieved through learning peers’ illness experiences which acted as a ‘living testimony’ that provided them with a sense of social validation and ‘made FMS visible’ [43, 50, 51]. Moreover, external recognition from the biomedical field legitimized FMS by providing care tailored towards the needs of patients with FMS [43, 45].

Internal legitimization and self-acceptance were shown to increase PA. When patients start perceiving their condition as a legitimate health problem, their willingness and confidence in making an effort to self-manage increases [43]. This is often prompted by acknowledging the clinical value of FMS, which facilitated their motivation to be actively involved in managing their condition [43, 45]. Furthermore, diagnostic clarification also facilitated internal legitimization, thus increasing PA by motivating patients to gain knowledge and maximize capability in self-management [45, 47]. Some patients also reported that limited information was provided post-diagnosis [46]. This then provoked them to actively seek information from other sources or participate in intervention programmes, which rewarded them with the necessary knowledge and skills [44, 50].

#### Value of medical support

Well-provided HCP support benefits the management journey and enhances PA of people living with FMS. An empathetic approach by HCPs was valued as patients’ confidence in self-managing increased when HCPs understood and addressed their needs [44, 49]. On the contrary, frustration and a lower willingness to self-manage were often caused by the incompetence of HCPs that lacked knowledge in FMS which overlooked patient’s specific needs [43, 49]. Moreover, the roles of HCPs in maximizing patient outcomes and the perceived effectiveness of intervention programmes were also identified. By addressing individual needs, managing expectations and providing professional advice, patients were motivated to engage in self-management interventions and perceived them as ‘effective’ [43, 45, 49]. Besides, individualized support from HCPs in aiding the identification of barriers in self-management facilitated patients to gain knowledge in developing coping strategies and overcoming obstacles [44–46].

Multi-component intervention programmes were positively regarded as ‘learning opportunities’ that aided the process of gaining knowledge and skills and facilitated efficient self-management [43–45]. Self-efficacy and motivation to engage in self-management techniques were also enhanced when the programmes gave participants a sense of purpose and motivation to implement the techniques they learned [43, 45]. These programmes also acted as a platform for peer support, where people with FMS were encouraged to interact with each other and share experiences during group sessions [43–45]. Moreover, adequate continued care is necessary to maintain PA and effective self-management over the illness course [43, 45, 46]. The time constraint in primary care sectors and the lack of follow-up sessions after secondary care provision were both shown to limit patients’ knowledge and ability to manage FMS [43, 45, 46, 48].

#### Receiving peer and social support

Many reported that a general lack of understanding from others and unsupportive family magnified the difficulty in self-management and formed negative experiences [48, 49]. On the contrary, social support was shown to improve self-efficacy and confidence in patients when their capability to engage in self-management was enhanced [44, 49]. Moreover, most patients established ways to efficiently self-manage by seeking help from others and considered it as a way to pace [48, 51].

Moreover, patients appreciated communicating with peers and commented that peer support ‘filled the gap in social understanding’ [43]. Peer support benefitted self-management by increasing internal readiness to exercise and participate in intervention programmes that legitimized FMS and improved PA [43–45, 50]. Since many experienced difficulties in social contexts and often experience isolation, social engagement was considered an important component of self-management [45]. Peer support was also found to improve patients’ social initiative and their confidence in social contexts, which indicated their increased activation in managing social situations [43, 50].

The effect of social comparison among peers was also found to improve PA [43, 50]. Downward comparison where meeting others with more severe symptoms motivated patients to take better care of themselves [50]. Social comparison also facilitated self-realization by noticing ‘I (the patient) am not handling it (FMS) as bad as I thought’ which increased patient’s confidence to implement self-management techniques [43].

#### Learning to self-manage

Four subthemes were identified—‘symptoms-driven information seeking’, ‘pacing techniques’, ‘negative past experiences’ and ‘illness duration’. These aspects of one’s illness experience illustrated their learning journey—from acquiring and implementing to accumulating knowledge and skills required to manage FMS. To begin with, although the nature of FMS and its interlinked symptoms caused difficulties in self-management, it also provoked patients to proactively seek information regarding treatment options and self-management techniques [47–49]. Therefore, with active information seeking, patients were able to enhance their capability and skills in self-management.

Implementation of skills thus comes after gaining knowledge. Among various self-management techniques, pacing-related skills such as prioritizing activities and identifying the ‘boom-bust’ pattern were found to have a more direct effect on PA [45, 46, 49]. Pacing techniques facilitated the identification of links between certain activities and fatigue levels, which enhanced their motivation and a sense of ‘regaining control’ [46]. Self-identification hence increased their knowledge, skills and capability to manage symptoms and activities more efficiently [45].

On the contrary, the results of engaging in physical activities often formed negative experiences in their self-management journey [48, 49]. The consequences of exercise, such as immense fatigue and pain, led to decreased PA. Negative past experiences regarding structured exercise programmes hindered their perceived capability to perform physical activities, thus resulting in an avoidant attitude towards exercises [48, 49].

In the context of PA and self-management, illness duration indicates the accumulation of knowledge and skills over time, making patients more proficient at self-management [47, 51]. By experimenting with different self-management skills and symptom-alleviating remedies, patients, therefore, take control over their health processes and are able to self-manage more efficiently and effectively [51].

## Discussion

### Discussion of findings

The findings of this review revealed four main aspects influencing PA and its relationship to effective self-management. Despite the idea of self-management being patients actively involved in managing their health, the findings shed light on the effects external impacts have on self-management and the interrelationships between external influence and one’s internal activation.

These findings aligned with a recent systematic review identifying similar internal and external factors influencing the self-management of chronic musculoskeletal pain [36]. Themes such as the importance of receiving external support from peers and social networks, as well as internal facilitators such as self-efficacy, were identified across studies in chronic pain, suggesting that these factors apply to the wider cohort of patients with chronic pain [16, 17, 36, 52–54].

This review highlights the role of HCPs facilitating PA. A study exploring the factors associated with PA in IA identified two key modifiable factors: self-efficacy and health literacy [17]. Our themes highlighted the need for information and expectation of individualized treatment. Participants valued psychological support from HCPs, which helped them develop independence by mastering their illness experiences, and fostering self-efficacy and PA [17, 54, 55]. Participants often took initiative and practiced self-management skills independently despite ongoing symptoms. However, unsupervised trial-and-error processes led to more negative experiences, highlighting the lack of HCP input and the need to improve health literacy [56–58]. Other shortcomings of current practice were also reflected in the findings. As this review focused solely on the illness experience of FMS, the importance of legitimizing the condition was accentuated. While self-acceptance is a key facilitator of self-management in chronic pain, the invisibility and invalidation of FMS may increase the need for external legitimization and diagnostic clarification [54, 59, 60].

To further conceptualize our findings, the developmental model of activation by Hibbard *et al*. [61] and the COM-B model by Michie *et al.* [62] were applied. The developmental model of activation formed the basis of the main concept of PA, which illustrated four stages—starting with an individual believing in the importance of their role in health promotion, thus having the confidence and knowledge necessary to take action, which progresses to actually taking health-promoting actions and ends with a sustained implementation of actions. Moreover, the COM-B model is a framework used to understand influences on self-management behaviour. The COM-B model identified three necessary components for any behaviour to occur: capability, motivation and opportunity.

According to the activation model, patients’ own beliefs regarding the importance of their role act as a starting point [61]. The significance of the theme ‘legitimizing FMS’ can therefore be explained. It was shown that via legitimization, patients are enabled to recognize FMS and its symptoms as ‘real’ [43]. Subsequently, the subtheme ‘diagnosis clarification’ further facilitated the identification of patients’ role in self-management. Patients viewed receiving the diagnosis as a starting point that guided them to identify the need to take an active role in managing the condition [63].

However, wider evidence indicated that some patients struggle to accept the diagnosis and reported a sustained low legitimacy in their illness experiences, alongside difficulties in self-management [64, 65]. Therefore, as recognized in the theme ‘value of medical support’, the role of clinicians in providing knowledge regarding the diagnosis is paramount in facilitating patients’ initial self-recognition of their role in the illness journey [23, 47].

Furthermore, this hierarchical activation process indicated that the further stages of implementing and maintaining behaviours require a sustained level of knowledge, skills and confidence [61]. This may explain the negative impact of inadequate continuous medical support on the ability to sustain health-promoting actions [66]. Studies identified that patients commonly report difficulties in maintaining newly learnt knowledge over time when there is a lack of follow-up sessions to support practising the implementation of skills [43, 45, 46].

However, the theme ‘learning to self-manage’ seemed to be less relevant or influential when applying the activation model. It may be due to the unidimensional nature of the model that illustrated the development of activation as a rather linear process where an individual progresses through stages [61]. As suggested by Jensen *et al.* [67], engaging in self-management and the readiness to adapt to changes may be multidimensional in nature, and it can be better reflected as continuous rather than through discrete stages [68]. The significance of this theme would be manifested when considering the COM-B model [62].

Capability, motivation and opportunity were identified as the essential components for behaviour change in the COM-B model. It also suggested that implementing behaviour changes can further increase or decrease one’s motivation or capability, forming a cycle [62]. This may explain how behaviours such as pacing techniques motivate patients and enhance their ability to self-manage. For instance, the study by Pearson *et al*. [46] indicated that their motivation increased when patients could regain control of their symptoms and self-management through pacing. On the contrary, negative experiences in implementing self-management behaviours were shown to negatively impact capability and motivation [48]. This is often seen in patients due to the unpredictability of FMS and its fluctuating symptoms; physical consequences of activities reduce patients’ capability to further engage with self-management tasks and thus their motivation and willingness to engage [49, 69].

The opportunity component can be considered a prerequisite for PA and self-management [70]. According to Michie *et al.* [62], ‘opportunity’ encompasses social aspects such as interpersonal influences and social cues. Therefore, external legitimization can be explained as providing social validation to patients and improving their social perception [43], thus improving one’s social initiative by being able to effectively communicate their needs to others and aid in self-management [50]. While the activation model mostly fits into explaining the development of PA, the COM-B model appears to explain the relationships between the factors, PA and self-management.

### Clinical implication

Our review indicated the importance of empathetic support from HCPs and the need for professionals to acknowledge the invisibility of FMS and legitimize the condition. HCPs working within a multi-disciplinary team should be aware of the factors that may affect adherence to intervention programmes and strategies that can be implemented to target these influencing factors. For example, shared decision-making and psychologist-led consultations may enhance acceptance and self-understanding, improving self-efficacy in functional activities [71–73] Pain and fatigue are often a concern for people with FMS engaging with physical activity [49]. Therefore, physiotherapy and occupational therapy interventions should adopt a personalized approach to balancing daily activities and physical activity. To facilitate family support, education to carers and support the development of health literacy and empathy should also be considered as an objective in the provision of care. Moreover, NHS-led interventions could be linked with peer support organizations to provide ongoing social support when needed [74].

### Study limitations

The review’s findings should be interpreted with the acknowledgement of the following limitations. Although the breadth of findings was aimed to be enhanced by including both the patient and HCP perspectives, it may be limited by the characteristics of some included studies. The participants in five of nine included studies were recruited from previous rehabilitation programmes, where it is likely that those who had negative experiences with the programmes generally opted out of the follow-up interviews or focus groups [45]. Although most studies reasonably interpreted diverse cases, the proportion of negative views may be different. Moreover, since the initial search trial including PA in the search terms did not yield many results, there is a risk of missing seminal papers by not including PA in the search terms, excluding mixed methods studies that are not in English and not searching grey literature.

### Future research

Sociodemographic differences were shown to correlate with the levels of PA [18]. Future studies may explore the differences in barriers and facilitators of PA across a wider range of socioeconomic communities. Additionally, a quantitative study on PA in FMS could use the Patient Activation Measure (PAM-13) to assess the impact of these identified factors on PA [13]. Future qualitative studies exploring patients’ perspectives on PA and using PAM to assess of their ‘activation level’ could further evaluate the suitability of the term ‘patient activation’.

## Conclusion

While self-management involves patients’ engagement, these findings highlight how external factors influence self-management and internal activation. Adequate support from HCPs emerged as key in facilitating the development of PA. The invisible nature of FMS increased the need for diagnostic clarification and legitimization from professionals. Clinicians involved in managing people with FMS should recognize potential barriers in care and prioritize providing effective patient education, displaying empathy and offering personalized support to ensure optimal outcomes.

## Supplementary Material

rkaf025_Supplementary_Data

## Data Availability

The authors agree to make materials, data and associated protocols promptly available to readers if requested.
