# Supplementary material for: Exploring patient activation and self-management experiences in adults with fibromyalgia: a qualitative evidence synthesis
Source: Rheumatol Adv Pract. 2025 Mar 10;9(2):rkaf025. doi: 10.1093/rap/rkaf025 (PMC11908766; doi:10.1093/rap/rkaf025)
Supplement: rkaf025_Supplementary_Data [file rkaf025_supplementary_data.zip › 08.07.2024 FMS paper ENTREQ.docx]

ENTREQ reporting checklist

| **Item** | **Guide and description** | **Reported in** |
| --- | --- | --- |
| Aim | State the research question the synthesis addresses. | Section 1 |
| Synthesis methodology | Identify the synthesis methodology or theoretical framework which underpins the synthesis, and describe the rationale for choice of methodology *(e.g. meta-ethnography, thematic synthesis, critical interpretive synthesis, grounded theory synthesis, realist synthesis, meta-aggregation, meta-study, framework synthesis).* | Section 2.6 |
| Approach to searching | Indicate whether the search was pre-planned (*comprehensive search strategies to seek all available studies)* or iterative (*to seek all available concepts until they theoretical saturation is achieved)*. | Section 2.2 |
| Inclusion criteria | Specify the inclusion/exclusion criteria *(e.g. in terms of population, language, year limits, type of publication, study type).* | Section 2.3 |
| Data sources | Describe the information sources used (e.g. *electronic databases (MEDLINE, EMBASE, CINAHL, psycINFO, Econlit), grey literature databases (digital thesis, policy reports), relevant organisational websites, experts, information specialists, generic web searches (Google Scholar) hand searching, reference lists)* and when the searches conducted; provide the rationale for using the data sources. | Section 2.2 |
| Electronic Search strategy | Describe the literature search *(e.g. provide electronic search strategies with population terms, clinical or health topic terms, experiential or social phenomena related terms, filters for qualitative research, and search limits)*. | Section 2.2 |
| Study screening methods | Describe the process of study screening and sifting *(e.g. title, abstract and full text review, number of independent reviewers who screened studies).* | Section 2.3 |
| Study characteristics | Present the characteristics of the included studies *(e.g. year of publication, country, population, number of participants, data collection, methodology, analysis, research questions).* | Section 3.2 |
| Study selection results | Identify the number of studies screened and provide reasons for study exclusion *(e,g, for comprehensive searching, provide numbers of studies screened and reasons for exclusion indicated in a figure/flowchart; for iterative searching describe reasons for study exclusion and inclusion based on modifications t the research question and/or contribution to theory development).* | Section 3.1, figure 1 |
| Rationale for appraisal | Describe the rationale and approach used to appraise the included studies or selected findings *(e.g. assessment of conduct (validity and robustness), assessment of reporting (transparency), assessment of content and utility of the findings).* | Section 2.4 |
| Appraisal items | State the tools, frameworks and criteria used to appraise the studies or selected findings *(e.g. Existing tools: CASP, QARI, COREQ, Mays and Pope; reviewer developed tools; describe the domains assessed: research team, study design, data analysis and interpretations, reporting).* | Section 2.4 |
| Appraisal process | Indicate whether the appraisal was conducted independently by more than one reviewer and if consensus was required. | Section 2.4 |
| Appraisal results | Present results of the quality assessment and indicate which articles, if any, were weighted/excluded based on the assessment and give the rationale. | Section 3.3 |
| Data extraction | Indicate which sections of the primary studies were analysed and how were the data extracted from the primary studies? *(e.g. all text under the headings “results /conclusions” were extracted electronically and entered into a computer software).* | Section 2.6 |
| Software | State the computer software used, if any. | Section 2.6 |
| Number of reviewers | Identify who was involved in coding and analysis. | Section 2.6 |
| Coding | Describe the process for coding of data *(e.g. line by line coding to search for concepts).* | Section 2.6 |
| Study comparison | Describe how were comparisons made within and across studies *(e.g. subsequent studies were coded into pre-existing concepts, and new concepts were created when deemed necessary).* | Section 2.6 |
| Derivation of themes | Explain whether the process of deriving the themes or constructs was inductive or deductive. | Section 2.6 |
| Quotations | Provide quotations from the primary studies to illustrate themes/constructs, and identify whether the quotations were participant quotations or the author’s interpretation. | Section 3.4 |
| Synthesis output | Present rich, compelling and useful results that go beyond a summary of the primary studies (e.g. *new interpretation, models of evidence, conceptual models, analytical framework, development of a new theory or construct).* | Section 3.4.1-3.4.4 |
