# Supplementary material for: Exploring patient activation and self-management experiences in adults with fibromyalgia: a qualitative evidence synthesis
Source: Rheumatol Adv Pract. 2025 Mar 10;9(2):rkaf025. doi: 10.1093/rap/rkaf025 (PMC11908766; doi:10.1093/rap/rkaf025)
Supplement: rkaf025_Supplementary_Data [file rkaf025_supplementary_data.zip › 24-148 Supplementary Material.docx]

# ***Supplementary materials***

Supplementary Table S1 – Results of Critical Appraisal Skills Program (CASP) appraisal

| # | Author | 1. Clear aim | 2. Method | 3. Research design | 4. Recruitment strategy | 5. Data collection | 6. Researchers-participants relationship | 7. Ethical issues | 8. Data analysis | 9. Statement of findings | 10. Value of research | Total score |
| --- | --- | --- | --- | --- | --- | --- | --- | --- | --- | --- | --- | --- |
| 1 | Arfuch et al. (2022) | 2 | 2 | 2 | 2 | 2 | 2 | 2 | 2 | 2 | 2 | 20 |
| 2 | Chen (2016) | 2 | 2 | 2 | 2 | 2 | 0 | 2 | 2 | 2 | 2 | 18 |
| 3 | Lempp et al. (2009) | 2 | 0 | 2 | 2 | 2 | 2 | 2 | 2 | 2 | 1 | 17 |
| 4 | Mcllroy et al. (2022) | 2 | 2 | 0 | 2 | 2 | 2 | 1 | 2 | 2 | 2 | 17 |
| 5 | Rasmussen, Amris and Rydahl-Hansen (2017) | 2 | 2 | 2 | 2 | 2 | 2 | 2 | 0 | 2 | 2 | 18 |
| 6 | Russell et al. (2018) | 2 | 2 | 2 | 2 | 2 | 2 | 2 | 2 | 2 | 2 | 20 |
| 7 | Sallinen, Kukkurainen and Peltokallio (2011) | 2 | 2 | 2 | 2 | 2 | 2 | 2 | 2 | 1 | 1 | 18 |
| 8 | Kengen Traska et al. (2011) | 2 | 2 | 1 | 2 | 2 | 0 | 2 | 2 | 2 | 2 | 17 |
| 9 | Pearson et al. (2020) | 2 | 2 | 0 | 2 | 2 | 1 | 2 | 2 | 2 | 2 | 17 |

(2 = Yes, 1 = Unable to determine, 0 = No)

Supplementary Table S2 – Results of Consolidated Criteria for Reporting Qualitative Research (COREQ) checklist

| **#** | **Item** | **Arfuch et al. (2022)** | **Chen (2016)** | **Lempp et al. (2009)** | **Mcllroy et al. (2022)** | **Rasmussen, Amris and Rydahl-Hansen (2017)** | **Russeel et al. (2018)** | **SallinenKukkurainen and Peltokollio (2011)** | **Kengen Traska et al. (2011)** | **Pearson et al. (2020)** | **No. Of studies** |
| --- | --- | --- | --- | --- | --- | --- | --- | --- | --- | --- | --- |
| **Domain 1: Research team and reflexivity** | | | | | | | | | | | |
| Personal Characteristics | | | | | | | | | | | |
| 1. | Interviewer/facilitator | X |  |  | X | X | X | X | X | X | 7 |
| 2. | Credentials | X | X |  |  | X |  |  | X |  | 3 |
| 3. | Occupation | X |  |  | X | X |  |  | X |  | 4 |
| 4. | Gender |  |  |  | X |  | (only specified gender of interviewer) | X |  |  | 2 |
| 5. | Experience and training | X |  | X |  | X | X |  |  |  | 4 |
| Relationship with participants | | | | | | | | | | | |
| 6. | Relationship established | X |  |  | X | X |  |  | X |  | 4 |
| 7. | Participant knowledge of the interviewer | X |  |  | X | X |  | X | X |  | 5 |
| 8. | Interviewer characteristics | X |  | X | X | X |  |  |  | X | 5 |
| **Domain 2: study design** | | | | | | | | | | | |
| Theoretical framework | | | | | | | | | | | |
| 9. | Methodological orientation and Theory | X | X | X | X | X |  | X |  | X | 7 |
| Participant selection | | | | | | | | | | | |
| 10. | Sampling | X | X | X | X | X | X | X |  | X | 7 |
| 11. | Method of approach | X | X | X | X |  | X | X | X | X | 8 |
| 12. | Sample size | X | X | X | X | X | X | X | X | X | 9 |
| 13. | Non-participation | X |  | X |  | X |  |  |  |  | 3 |
| Setting | | | | | | | | | | | |
| 14. | Setting of data collection | X | X | X | X | X | X | X | X | X | 9 |
| 15. | Presence of non-participants | X |  |  | X | X | X |  | X | X | 6 |
| 16. | Description of sample | X | X | X | X | X | X | X | X | X | 9 |
| Data collection | | | | | | | | | | | |
| 17. | Interview guide | X | X | X | X | X | X | X | X |  | 7 |
| 18. | Repeat interviews |  | X |  |  | X |  |  |  |  | 2 |
| 19. | Audio/visual recording | X |  |  | X |  | X | X | X | X | 6 |
| 20. | Field notes | X |  |  | X | X | X | X | X | X | 7 |
| 21. | Duration | X | X | X | X | X | X |  |  | X | 7 |
| 22. | Data saturation | X |  | X |  | X | X |  |  |  | 4 |
| 23. | Transcripts returned | X |  |  | X |  | X |  |  |  | 3 |
| **Domain 3: analysis and findings** | | | | | | | | | | | |
| Data analysis | | | | | | | | | | | |
| 24. | Number of data coders | X |  | X | X | X | X |  | X | X | 7 |
| 25. | Description of the coding tree |  |  |  |  | X |  |  | X |  | 2 |
| 26. | Derivation of themes | X | X | X | X | X | X | X | X | X | 9 |
| 27. | Software | X | X | X | X |  |  |  |  | X | 5 |
| 28. | Participant checking | X |  |  |  |  |  | X |  |  | 2 |
| Reporting | | | | | | | | | | | |
| 29. | Quotations presented | X | X | X | X |  | X | X | X | X | 8 |
| 30. | Data and findings consistent | X | X | X | X | X | X | X | X | X | 9 |
| 31. | Clarity of major themes | X | X | X | X | X | X | X | X | X | 9 |
| 32. | Clarity of minor themes | X | X |  |  | X |  |  | X |  | 4 |
| Total | | 29 | 16 | 17 | 24 | 25 | 19 | 17 | 20 | 18 |  |

Supplementary Table S3 – Supporting evidence to each subtheme

| **Themes** | **Subthemes** | **Supporting evidence** |
| --- | --- | --- |
| Legitimising FMS | Internal legitimisation | A - While existing treatment strategies are essentially based on pharmacological symptom control and lifestyle changes, patients may not be willing to make any effort if they do not perceive their condition as a real and frequent health problem that requires being addressed to improve functionality and QOL and to prevent future complications. (Arfuch *et al.,* 2022, p.6)  A - Interviewees suggested that the proposed MCI contributed to providing clinical value to FMS and its patients, which encouraged them to adopt an active role in their health process (Arfuch *et al.,* 2022, p. 7)  P - *“The program represents the tranquility of having a point of reference. I used to think it was just my imagination or that I was a complaining person. However, it turns out that it is much more complex than that.” (Arfuch et al., 2022, p.7)*  A - As acceptance increased, participants experienced a sense of being strengthened, from being acknowledged by skilled professionals, leading to increased self-confidence (Rasmussen, Amris and Rydahl-Hansen, 2017, p. 939)  P - *“… I adjusted to a year of concentrating on wellness, instead of just, “Oh, I’m going to be...cured.” Because I kind of felt like, “I’m going to cure myself!”...I think it’s kind of like a pipe dream that some people like me will cling to and...I need to accept...I think you have to grieve like you have to grieve any other loss or death...you have to go, “Okay that’s the old me,” and “this is the new me.” (Chen, 2016, p. 5)* |
|  | External legitimisation | A - While there is no medical evidence for supporting FMS, it has been detected that peers’ illness experiences provide living testimony to support this health condition. Hence, the significant other seems to play a key role in making sense of one’s health experience and promoting the sense of belonging to a group. (Arfuch *et al.,* 2022, p. 7)  A - According to our informers, the MCI legitimized FMS by validating patients’ illness accounts. Patients reported a sense of being cared about given the provided health education, psychological guidance, professional support, and continuous follow-up (Arfuch et al., 2022, p.5)  P - *“All the information I got was good, of course. . . but I think it was this talking with others... you see, I had said earlier [before diagnosis] that fibromyalgia is an ‘‘illness’’ in inverted commas, not a real one... and it was difficult for me to change this perception. . . but there I began to understand better. . . discussions with the others...those were really important... these symptoms are real, and not just something imaginary. . .”* (*Sallinen, Kukkurainen and Peltokollio, 2011, p. 128*)  P - *“I thought that it is good to see others… to hear how they have managed with things and how they have treated themselves… and I got all this… I learned how FMS is studies widely all over the world and that it really is a disease… not just something obscure… and that there are practical things that help you cope with it…” (Sallinen, Kukkurainen and Peltokollio, 2011, p. 128)* |
|  | Diagnosis clarification | A - Nevertheless, being diagnosed was important because the diagnosis enabled participants to move forward in terms of figuring out how to manage their condition. (Chen, 2016, p. 5)  A - Once participants had an idea that fibromyalgia was what they had, they would engage in more extensive information seeking. (Chen, 2016, p. 7)  A - Some participants found the diagnosis empowering in that it enabled them to do something to help themselves. (Chen, 2016, p. 7)  A - Almost all patients (11/12) critically remarked that very little or no information was given to them following their FMS diagnosis. This required the interviewees to be proactive in seeking information from other sources, such as the internet, leaflets, self-help groups or books. (Lempp *et al.,* 2009, p.4)  A - Participants also expressed the significance receiving diagnostic clarifications, and being confirmed in that the condition is physical, so that they stopped doubting themselves and were reassured that they were not about go crazy. (Rasmussen, Amris and Rydahl-Hansen, 2017, p. 939) |
| Value of medical support | Intervention programmes | A - In this regard, the MCI program proved to provide patients with a sense of purpose and motivation to carry out new plans. Informers shared their experiences on taking new courses, keeping busy, and exercising daily (Arfuch *et al.,* 2022, p. 10)  A - FAME was recognised as a learning opportunity for most participants. Patient participants learnt about their condition, the spectrum of symptoms and new ways of managing these. (Mcllroy *et al.,* 2022, p. 691)  A - They reported that they would like more exercise within FAME. One patient participant reported that FAME taught everyone to exercise – *“in the best way that suits you and [that you] were comfortable whilst doing the exercises. So that was really helpful.”* (Mcllroy et al., 2022, p. 692)  A - Although they were concerned that it could be hard to maintain the new knowledge over time, due to the lack of follow-up sessions. (Rasmussen, Amris and Rydahl-Hansen, 2017, p. 939) |
|  | HCP support | A - Patient participants views on the support provided by the HCPs varied. Some patient participants reported HCPs worked with/ alongside them to understand and address any problems they had and that this increased their self‐efficacy to self‐manage. *“I was thinking that I was, I would be able to sort my problem because he was beside me” (Mcllroy et al., 2022, p. 691)*  A - In this regard, informers described hopelessness when their proprioceptive experience is overlooked by the biomedical field and the social network. Further discernment revealed how patients could have undergone emotional dejection, confusion, and doubts regarding their suffering, which led to a nihilistic attitude towards themselves and the healthcare services. (Arfuch *et al.,* p. 6)  A - However, participants experience an existential strain in their efforts to acknowledge and accept living with the disease, and many were lacking individual consultations with the psychologist, to further support them in their acknowledgement and acceptance. (Rasmussen, Amris and Rydahl-Hansen, 2017, p. 938)  A - Some patients found the breadth topics and the vernal nature of the content did not meet their needs and wanted more individual support (Mcllroy *et al.,* 2022, p. 691)  A/P - Participants reported that understanding and empathy for the sufferers and their personal situation were important, and would enable any treatment to be more effective: PT8: “*somebody who understands and has empathy for you and so you don’t feel judged or pushed or pressured. Someone who just gives you a bit of empathy and doesn’t just say to you, ‘could you not keep trying?’ because I would if I could. I don’t need you to tell me to keep trying… just a bit of understanding and empathy would be a great start.”* (Russell et al., 2018, p. 510) |
| Receiving peer and social support | Peer support | A - The group effect was found to benefit patients’ self-confidence in social contexts as well as their social initiative. Analytically, as peers become significant others, patients’ self-worth grows and their social performance anxiety and low social perception decrease. (Arfuch *et al.,* 2022, p.7)  A - The peers also gave practical advice for managing the difficulties in everyday life, such as how to deal with sleeping problems or how to cope with constant pain. (Sallinen, Kukkurainen and Peltokollio, 2011, p. 128)  A - The participants experienced that their own acceptance of living with FMS was strengthened, and that their ability to be acknowledging themselves with the illness was strengthen from identifying with the others patients in the group, with regard to how the disease impacted on their functional ability (Rasmussen, Amris and Rydahl-Hansen, 2017, p. 938) |
|  | Social support | A - Participants felt strengthened through support from the others in the group and increased support from their significant others, and they felt that they could tolerate more (Rasmussen, Amris and Rydahl-Hansen, 2017, p. 939)  A - Patients found ways of managing their lives by making compromises and by sharing activities with the help of supportive family members and friends (Lempp *et al.,* 2009, p.7) |
|  | Comparing with others | A - On the other hand, meeting others with more severe symptoms was seen as a motivation for taking better care of oneself (Sallinen, Kukkurainen and Peltokollio, 2011, p. 129)  P - *“… There were many who were in really bad condition and who had problems with mental health. . .I could not help thinking if that is to be my destiny, too. . . of course sometimes when you are really … [in pain] ....you find yourself thinking when walking along the riverbank. . .how easy it would be to jump into the water. . .but then you just think of all the good things you have. . . I have decided that I will not let it get so bad that it wipes me out. . .I have managed to struggle and win so far…” (Sallinen, Kukkurainen and Peltokollio, 2011, p. 129)*  A - However, this experience was helpful to find out that there are many people in worse conditions than me. In this sense, I realised that I am not handling it as bad as I thought (Arfuch *et al.,* 2022, p.8)  P - *“Many times, you feel bad, but maybe next to you there is another person who is feeling worse than you. And then you think: “well, maybe my situation is not so terrible”. And this strengthens you and gives you a little more encouragement” (Arfuch et al., 2022, p. 9)* |
| Process of learning to self-manage | Symptoms-driven information seeking | A - First, because information seeking was often symptom-driven, as participants were able to get their symptoms under control and/or learned to manage their condition better, they felt less of a need for information. (Chen, 2016,, p. 7)  P - *“If you have any intelligence at all and you want to get better, you want to try to figure out why you woke up feeling so bad. So I would go back and go through all the foods that I ate and go through everything. Did I go through these stresses?” (Chen, 2016, p. 9)* |
|  | Negative past experiences | A - These consequences of exercise led to avoidance of exercise *“exercise to me is a scary word”* (PT8), and the change in attitude regarding what activities were or were not achievable. (Russell et al., 2018, p. 511)  A - The physical symptoms that resulted from exercise shaped the attitudes and feelings associated with exercise and physical activity. (Russell et al., 2018, p. 511) |
|  | Pacing techniques | A - Activities that resulted in increased levels of fatigue, subsequent sustained low levels of physical activity and inactivity were identified. (Pearson *et al.,* 2020, p. 376)  Patients who engaged in this activity found that the act of creating a visual summary of their activity patterns helped them to identify negative behaviours (Pearson *et al.,* 2020, p. 377)  A - The therapists explained that pacing is given as a way of changing their internal and external motivation by teaching patients that rather than being controlled by symptoms, pacing offers a way to regain control (Pearson *et al.,* 2020, p. 379)  A - Although many reported that their mind wanted to do more than their body could perform, they were striving to adapt performance of activities to their resources to avoid exhaustion, and to perform the relaxation exercises, introduced in the programme. Participants expressed that they were learning to adapt the pace of activity execution to their abilities when performing daily life activities (Rasmussen, Amris and Rydahl-Hansen, 2017, p. 940) |
|  | Illness duration | P - *“It took me a long time to be the manager of my own health system. I expected doctors to kind of manage my life for me. It took me a long time to realize that, no, I’m in charge of this.” (Chen, 2016, p. 8)*  P - *“For once in a very long time, I felt like I could handle the fibromyalgia...I was starting to realize: ok, it’s a part of my life, and I started noticing some of the triggers a little bit better, also the best way to manage some of the symptoms, and also not being so mad at myself or my body.” (Chen, 2016, p. 5)*  P - *“One indicated that over time, she has developed a variety of coping methods: “the longer it’s been, the more I’m able to - to know what it is i should do and i have topical ointments that i can use or i can take an extra pain pill or just, you know, lay down” (Traska et al., 2011, p. 630)* |

Note: First order constructs, text extracts from participants, are indicated with the letter “P. Second order constructs based from the authors’ interpretation are marked as “A”.
